# Institutional Questions

**Published:** 1901-02-02

**Authors:** 


### INSTITUTIONAL QUESTIONS.

### VISITORS FOR THE UNVISITED IN HOSPITALS.

A CORRESPONDENT writes: "I was a little disappointed at THE HOSPITAL paragraph on the subject of 'Visitors for the Unvisited in Hospitals,' because the verdict was given before the case was stated. I am sure you would be the last to oppose a plan for bringing voluntary hospital visitors into a sympathetic union. So far ladies and women in general have not taken the interest in hospital work which they should, and I am sure you will sympathise in any effort to rouse women's interest in our hospitals."

Our correspondent is evidently out of touch with the internal working and actual circumstances of the best hospitals in England to-day. So far as our knowledge and information go the movement to form an association of ladies to visit hospitals, arising, as it evidently does, rather from kindness of heart than a precise apprehension of the facts, is being organised on wrong lines. The truth is, that under the improved methods of hospital treatment the number of days during which a patient is resident at the present time in a hospital has been reduced to a comparatively brief period, and the length of that period is diminished by the circumstance that most well-organised hospitals have now attached to them convalescent institutions to which the patients are transferred immediately the stage of convalescence is reached. It follows that the large majority of the patients in the best-administered of our hospitals to-day are during their residence for the most part in the acute stage of their maladies, when visitors, being strangers, would be undesirable and in some cases positively harmful. Again, since the introduction of so many nurses, under trained and capable sisters, every patient has continuous attention, and there is plenty of diversion, owing to the active life always going on around him, whilst on the regular visiting days the large majority of the patients are visited by their friends. Thus it will be seen that there is little or no opening for a corps of hospital visitors in the sense which the association favours. What little opening there is is already supplied through the constituted officials and governors, in co-operation with the Samaritan or kindred societies, which are attached to the principal hospitals, and indeed to most hospitals nowadays. It is possible that such an association as is proposed might find a useful field for work in convalescent institutions, where we have reason to think many things go on which are not desirable, and which would probably be remedied were they to be regularly visited by capable persons, carefully selected so as to guarantee that they should invariably act judicially and never fail to co-operate with the officials. Here is a field for the introduction of talent by the organisation of suitable amusements and occupation for the large majority of the inmates, especially on dull days and in bad weather.

Generally, the League of Mercy, which has been incorporated by Royal Charter, embodies the association's idea, and carries it out so far as practicable by teaching the doctrine of personal service rendered in the first instance by individuals under a district organisation throughout the metropolis; and in the second by enabling those individuals to get in touch with the hospitals, to learn to understand their work and needs and to take a most useful part in alleviating the latter and helping in the former. We think, therefore, that the ladies who have expressed their willingness to join the movement in question, if they were made familiar with the work and objects of the League of Mercy, might usefully utilise their energies by becoming members of that League, and so render practical service to the hospitals under the highest and best auspices.

### PAY WARDS.

A CORRESPONDENT writes: The committee of a hospital possessing a building too large for its requirements, for a few years, possibly, proposes to have one or two private wards into which the members of the staff may send the private cases, these cases paying from one to three guineas per week for nursing and care in the hospital; those paying two guineas a week and over to be supplied with dressings and anæsthetics. It is thought the funds of the hospital would thereby be helped. Is there any professional or other objection to this step?

There can be no objection, in our view, to the establishment of a paying wing in connection with a general hospital admitting patients without payment, providing (*a*) that the pay wing is devoted entirely to pay patients; (*b*) that it has a separate approach and entrance, so that the medical attendants and the patients' friends do not enter the building devoted to non-paying patients; (*c*) that each patient can at his pleasure be attended by his own doctor at fees to be agreed; (*d*) that the amount received by the hospital from the patients is sufficient to defray all cost of his residence and to leave a small profit for the institution. We do, however, see that in a building purchased for public purposes and devoted to the treatment of non-paying patients, the appropriation of certain portions of that building, at the request of the medical staff, to paying patients, who would pay the staff full fees for their medical treatment, might be open to very serious abuses. That abuse would be greatly increased if the weekly sum paid by the paying patients to the hospital were only 30s., a sum which in any circumstances must be less than the outlay entailed upon the hospital by the residence of a patient in the pay ward.—ED. T. H.

